# Long Term Ortho-surgical Management of Temporomandibular Joint Ankylosis Secondary to Childhood Infection

**DOI:** 10.31661/gmj.v13iSP1.3675

**Published:** 2024-12-29

**Authors:** Hossein Behnia, Azita Tehranchi, Parsa Behnia, Navid Tariverdi, Abolhassan Mesgarzadeh

**Affiliations:** ^1^ Dental Research Center, Shahid Beheshti University of Medical Sciences, Tehran, Iran; ^2^ Dentofacial Deformities Research Center, Research Institute for Dental Sciences, Shahid Beheshti University of Medical Sciences, Tehran, Iran; ^3^ Department of Oral and Maxillofacial Surgery, Faculty of Dentistry, Shahed University, Tehran, Iran; ^4^ Department of Orthodontics, School of Dentistry, Shahid Beheshti University of Medical Sciences, Tehran, Iran; ^5^ Department of Oral and Maxillofacial Surgery, Faculty of Dentistry, Tehran University, Tehran, Iran

**Keywords:** Distraction Osteogenesis, Infection, Orthognathic Surgery, Temporomandibular Joint Ankylosis

## Abstract

This case report presents the management of a patient suffering from severe facial asymmetry and malocclusion. The patient was born in 2001 and was later hospitalized due to severe jaundice. At 5 years of age, the deviation in the jaws and the post condylectomy ankylosis of the TMJ and hip joint problems were evident. At age 7, a costochondral graft was performed. In the next phase, orthodontic treatment was started with hybrid functional therapy, but the treatment was unsuccessful due to the severity of the problem. At age 15, bilateral distraction osteogenesis was performed. The orthodontic plan for the maxilla was non-extraction. We expanded the upper arch and corrected the crossbite of the lateral incisors. Due to the difficulty of surgery, the impacted canine of the mandible remained impacted. The mandibular right lower incisor was removed due to severe crowding. Orthognathic surgery was performed after fixed orthodontic treatment.

## Introduction

The word ankylosis comes from Greek and means stiffening of joints. Ankylosis of the temporomandibular joint (TMJ) involves attachment of the condyle and glenoid fossa, disc, or eminence, often resulting in limited mandibular movements. In individuals with TMJ ankylosis, mouth opening is usually <6 mm [1]. Ankylosis of the joint also causes problems with feeding, speech, and oral hygiene [2]. In addition, TMJ ankylosis in childhood often has a detrimental effect on facial growth, ultimately leading to facial asymmetry, mandibular micrognathia, a Cl II skeletal relationship, and facial deformity [3]. TMJ ankylosis is more common in men than women, and the ratio of unilateral-to-bilateral ankyloses is reported to be approximately 1.5: 1. The most common cause of TMJ ankylosis is trauma, but it can also occur as a result of local and systemic infection, systemic diseases such as rheumatoid arthritis and psoriasis, or previous TMJ surgery.

Ankylosis of TMJ caused by infection is a well-known phenomenon. Topazian [4] found that infection is involved in 68% of TMJ ankylosis cases [5]. Septic arthritis is a purulent joint infection that occurs when microorganisms invade the joint space, either through the hematologic spread of a distant infection or direct inoculation of the joint [6]. Joint ankylosis can occur due to traumatic joint exposure to microbial invasion or odontogenic, ear, or skin infections. Since the joint synovium is vascularized and has no limiting basement membrane, it is vulnerable to hematologic infections. Most infections are monoarthritic. However, 10-20% are polyarthritic, with the knee being the most commonly involved joint. It rarely affects smaller joints, such as the TMJ [6]. These hematologic infections can also affect other joints, such as elbows, shoulders, and hips [7].

Early diagnosis and treatment are essential because septic arthritis of the TMJ may cause serious sequelae, including joint dysfunction, growth disorders, fibrosis, and ankylosis, or lead to the spread of infection to adjacent areas [6].

Management of TMJ ankylosis is usually surgery. Surgical procedures include gap arthroplasty (GA), interpositional gap arthroplasty (IGA), reconstruction arthroplasty (RA), and distraction osteogenesis (DO) [8][9]. Early diagnosis and treatment are essential [6].

## Case Presentation

**Figure-1:**
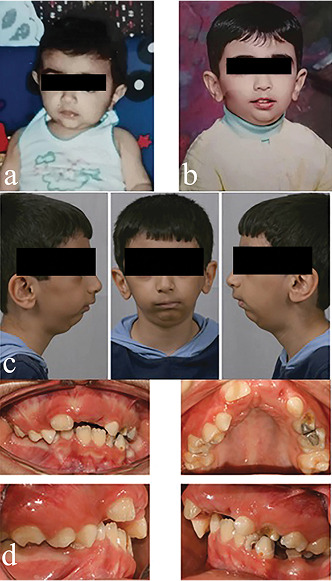
Figure 1.The patient before surgery.

**Figure-2:**
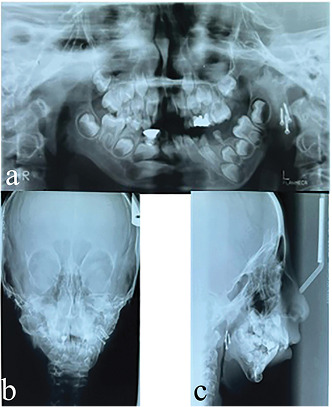
Figure 2.Panoramic, lateral, and PA cephalometric images after condylectomy and costochondral surgery.

History

A male patient born in 2001 was hospitalized due to severe jaundice after birth. When the child began to toddle, a difference was noted between the left and right legs. The parents did not report any history of trauma to the face and chin. In addition, there was no history of dental abscess or tumor in the head and neck region, and the delivery had been uneventful. The patient had ankylosis of the left condyle due to an unknown hematologic infection. The patient’s medical history was unremarkable.

Extraoral examinations revealed facial asymmetry with mandibular deviation in opening to the left, mandibular retrognathism, and barely noticeable facial elongation on the healthy side. TMJ examination revealed limited mouth opening—interincisal mouth opening was 6 mm—markedly limited external and lateral protrusive movements, and deviation of the mandible to the affected side after opening. The amount of overjet before the treatment was 9 mm. A panoramic view showed that the child had all permanent teeth, but the canine tooth on the right side of the mandible was in a horizontal and inappropriate position.

Treatment Objectives

At age 7 (2008), condylectomy and costochondral graft surgery were performed. Almost 8 mm of the length of the right fifth rib, along with the attached cartilaginous part, was removed for grafting. Figure 1 shows the patient before the surgical procedure.

Figure 2 shows the panoramic, lateral, and PA cephalometric images after condylectomy and costochondral grafting.

**Figure-3:**
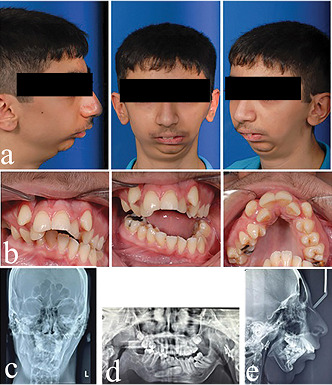
Figure 3. Pictures of the patient’s face, intraoral pictures and panoramic radiographs, lateral cephalometry, and PA cephalometry after functional treatment

After condylectomy, the patient was referred to an orthodontist for further treatment, and treatment was initiated with a hybrid functional appliance. The functional appliance encourages growth on the deviated side and prevents the formation of the cant of the occlusal plane. Unfortunately, the patient did not respond to treatment due to the severity of the problem (Figure 3). At age 15, asymmetric bilateral distraction osteogenesis was performed for the patient to reduce the discrepancy and jaw movements during surgery. Second molars of mandible were lost due to the DO procedure.

**Figure-4:**
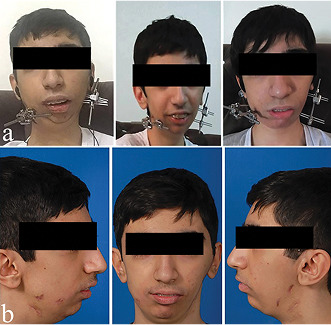
Figure 4. Images during DO treatment and after removing the appliance.

Figure 4 shows the patient during treatment and after removing the appliance.

**Figure-5:**
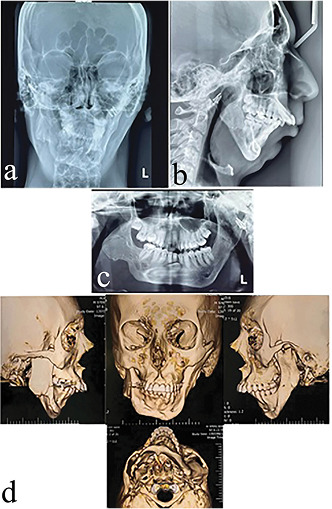
Figure 5.Panoramic and lateral and PA cephalometric images and CT scans before the start of fixed treatment.

**Figure-6:**
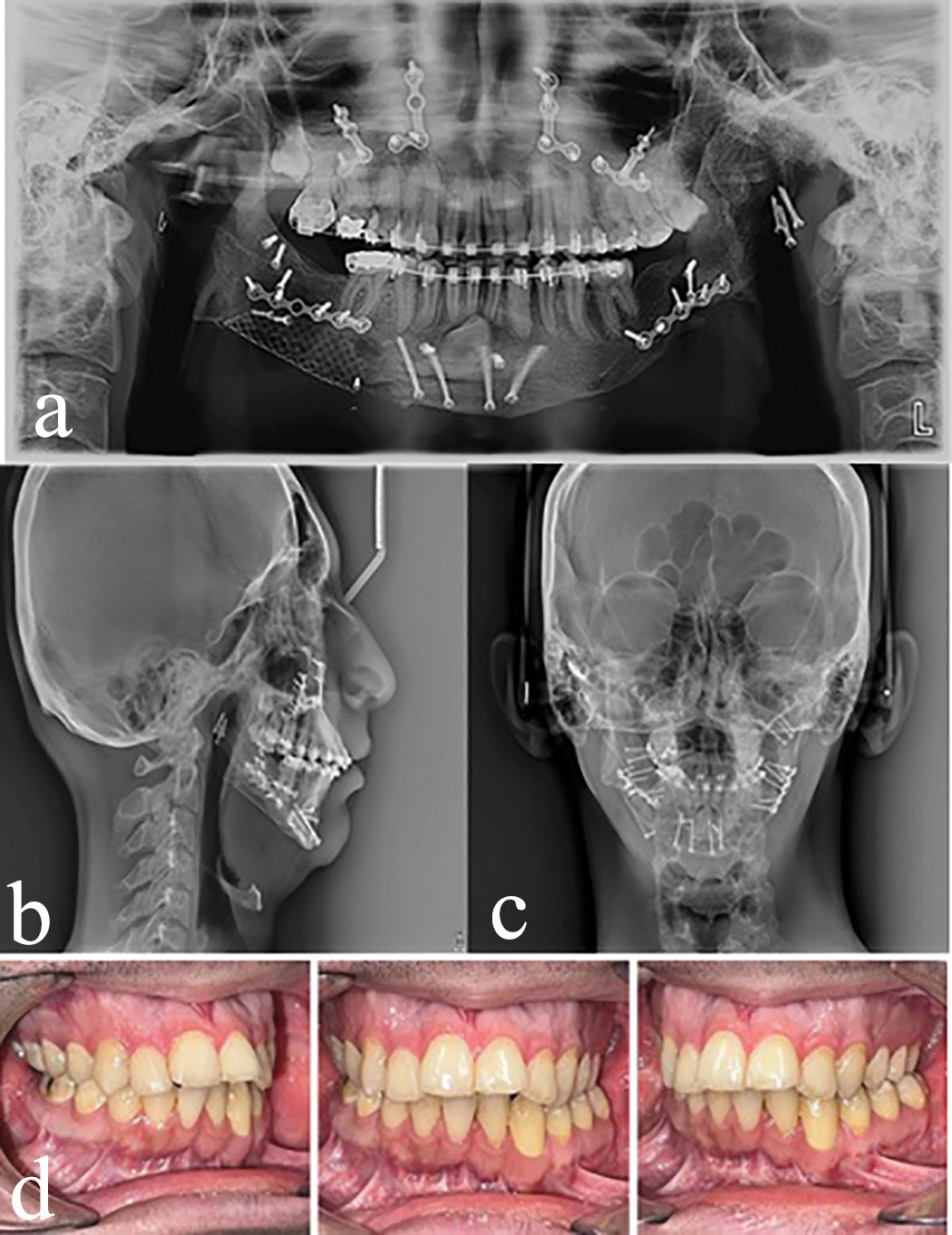
Figure 6. Panoramic views and lateral cephalometry, PA cephalometry, and intraoral images at the end of treatment.

In the upper arch, orthodontic treatment plan was non-extraction due to the proper torque of the anterior upper teeth and the discrepancy not being reduced compared to the lower jaw. We expanded the upper arch and corrected the crossbite of the lateral incisors. Orthognathic surgery was performed after fixed orthodontic treatment (Figures 5 and 6).

**Figure-7:**
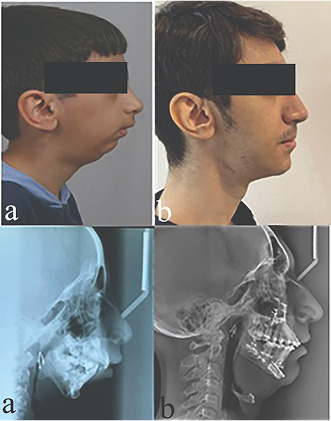
Figure 7.Before and after treatment.

**Figure-8:**
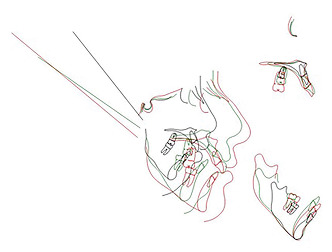
Figure 8.The patient before surgery.

As shown on radiographic images, the lower right canine tooth is impacted. Due to its horizontal position, it cannot be returned to its normal position due to the risks and injuries during surgery. The canine tooth remained in that position. Considering the severity of mandibular crowding, the mandibular right lower incisor was removed. During the fixed orthodontic treatment of the lower jaw, to prevent the resorption of the roots of the anterior teeth, they were not engaged, and arch coordination was performed. Finally, bimaxillary surgery was performed for the patient at 22 years of age. At the end of the treatment, the maximum mouth opening was 13 mm and overjet and overbite were 3 mm. Table-1 presents the results of cephalometric analyses. Figure 6 shows the results of the treatment. Figure 7 is a comparison between the periods before and after treatment. Figure 8 shows the superimposition of the patient at 7, 15, and 22 years of age based on cranial base adaptation. The patient was referred for the replacement of mandibular second molar with implants.

## Discussion

**Table:** Table1. The results of lateral cephalometric analyses of the patient at 7, 15, and 22 years of age

	5	15	22
**FH-SN**	1.9	1.8	2
**Saddle angle**	118.1	114.4	114.5
**Articular angle**	173	168.3	176
**Gonial angle**	140.7	151.1	129
**Sum of angles**	431.8	434.2	419.6
**y-axis**	94.3	90.9	84.1
**Palatal plan inclination**	73	81	77
**S-Go/N-Me**	48.6	45.8	49.5
**PP-MP**	49.4	60.8	36.1
**MP-SN**	71.8	74.2	59.6
**OCC Plane to SN**	38.4	31.3	31.5
**SNA**	66.2	70.4	72
**SNB**	57.6	61.6	69
**ANB**	8.6	8.7	3
**SN-NPog**	55.1	59.4	66
**Wits**	3.2	7.7	-1
**U1-SN**	85.4	109.8	91
**IMPA**	79.3	78.2	87
**N1-NA**	3.1	10	4.7
**L1-NB**	5.5	8.5	6
**Interincisal angle**	123.4	97.8	124
**U1-Palatal plane**	107.9	123.2	114
**Nasolabial angle**	114.7	113.4	112
**Soft tissue convexity**	130	118.7	129
**mandible**	32	55	65
**Maxilla**	30	39	43
**Ramus**	30	32	40

Ankylosis of the TMJ is a seriously debilitating disease that significantly impacts the patient’s quality of life, nutrition, speech, oral hygiene, growth, occlusion, and facial aesthetics. In severe cases, it can cause micrognathia, which can lead to obstructive sleep apnea. In children, unilateral ankylosis of the TMJ often results in facial asymmetry due to the deviation of the chin to the affected side, often with mandibular growth [10]. This complication can be classified based on the location (intra- or extra-articular), the type of tissue involved (osseous, fibrous, or fibro-osseous), or the degree of fusion (complete or incomplete) [11]. TMJ ankylosis is also classified as "true ankylosis," in which conditions (e.g., trauma, infection, or arthritis) cause bony or fibrous adhesions in the TMJ capsule or "false ankylosis" (also called pseudoankylosis) if joint movements are restricted due to pathology unrelated to the joint components, for example, muscle or nerve disorders [12].

TMJ ankylosis can be induced by various causes, such as trauma, degenerative disorders, infection, or space lesions [13].

Although condyles in children have a high regeneration ability, the rate of ankylosis increases significantly after trauma or surgery [14].

Local predisposing factors for the development of ankylosis include blunt trauma, previous joint disease (e.g., arthritis), and burn wounds. On the other hand, systemic predisposing factors include systemic and autoimmune diseases [15] (rheumatoid arthritis, Reteiter’s disease, alcohol abuse/hypogammaglobulinemia) [16], drugs [6] (e.g., systemic steroids), and sexually transmitted diseases. Trauma, infection, degenerative changes, rheumatic disease, iatrogenic causes, and space lesions can be associated with TMJ septic arthritis and are not often reported in neonatal patients [5]. The cause of these infections is often unknown, but they can spread through an open wound or the bloodstream [17]. This pathologic condition is very rare in infants [7].

Septic arthritis is a rare disease related to systemic and local factors such as the involvement of TMJ by rheumatoid arthritis or systemic factors such as immunosuppression, diabetes, or the long-term use of systemic steroids [18]. This infection usually occurs due to head and neck infections or through the transmission of infection from distant areas. The most common bacteria involved are Staphylococcus aureus, Neisseria gonorrhoeae, and Haemophilus influenza [12].

The most common Gram-negative organisms are Pseudomonas aeruginosa and Escherichia coli, which are usually reported in patients with a history of taking intravenous drugs, infants, the elderly, and immunocompromised patients [19].

Patients with septic arthritis of the TMJ usually show mandibular erythema and trismus, and the preauricular area is swollen. In addition, patients may have fever, weakness, malocclusion, and local lymphadenopathy. Forward deviation of the mandible during opening is a common clinical sign caused by increased joint fluid. Traumatic effusions, fractures, and neoplasms may appear, resembling septic arthritis [12].

Bacterial infection of TMJ causes increased synovial hyperplasia, necrosis, granulation tissue, and abscess formation. Proteolytic enzymes released by granulocytes may cause irreversible changes within seven days [20].

Several surgical techniques can be used to treat TMJ ankylosis in pediatric patients: coronoidectomy, bone distraction, gap arthroplasty, costochondral grafting, and total joint replacement. The timing and ideal sequence of different interventions are still debated [21][22]. The success of these methods is determined by maintaining maximum incisal opening, preventing re-ankylosis, and stimulating mandibular growth[23].

Management of TMJ ankylosis in children is very difficult due to the unpredictability of mandibular growth and the high prevalence of ankylosis. First, the child is referred to an anesthesiologist to check the airway. In our case, resection and reconstruction surgery with CCG was performed. Costochondral graft is generally considered the first choice for treating this disease in infants due to the ease of harvesting and the compatibility of the transplant, the low complications of the donor site, the biological and anatomical similarity with the mandibular condyle, and its growth potential [24]. The CCG should theoretically allow the healthy side to maintain the same growth pattern and mandibular symmetry throughout the patient’s growth process. However, graft growth can be uncontrolled and unpredictable, ultimately resulting in facial asymmetry that causes functional and esthetic defects. Long-term reports of mandibular growth in children with reconstructed TMJ using CCG indicate overgrowth on the treated side [25]. However, in our case, it was not very successful due to the severity of the problem and perhaps the patient not using the functional appliance. At the end of treatment, there was no translational movement due to the presence of fibrotic tissue in the TMJ.

## Conclusion

In conclusion, early diagnosis and surgical and orthodontic intervention of TMJ ankylosis are very important, especially in growing children. With early diagnosis, we prevent growth impairment, facial asymmetry, and mandibular growth restriction, which significantly impacts the child’s mental and physical health. Although there is currently no protocol in these cases, early treatment of ankylosis with no restriction of mandibular movements and orthodontically proper management of the patient, including the creation of stable occlusion, can be considered a successful treatment.

## Conflicts of Interest

The authors declare no conflicts of interest.
